# Slope-reducing tibial osteotomy combined with primary or revision ACL reconstruction improves knee stability and subjective function in patients with steep posterior tibial slope: a systematic review and meta-analysis

**DOI:** 10.1097/JS9.0000000000003507

**Published:** 2025-09-19

**Authors:** Daofeng Wang, Menglinqian Di, Tong Zheng, Hui Zhang

**Affiliations:** a Sports Medicine Service, Beijing Jishuitan Hospital, Capital Medical University, Beijing, China; b Beijing Research Institute of Traumatology and Orthopaedics, Beijing, China

**Keywords:** anterior cruciate ligament reconstruction, knee stability, meta-analysis, posterior tibial slope, slope-reducing tibial osteotomy

## Abstract

**Purpose:**

To systematically review and quantitatively analyze the clinical outcomes of slope-reducing tibial osteotomy combined with anterior cruciate ligament (ACL) reconstruction (ACLR) in patients with steep posterior tibial slope (PTS).

**Methods:**

This study was performed in line with PRISMA (Preferred Reporting Items for Systematic Reviews and Meta-Analyses) and AMSTAR (Assessing the Methodological Quality of Systematic Reviews) Guidelines. PubMed, Embase, Web of Science, and Scopus databases were searched to identify studies reporting clinical outcomes of slope-reducing tibial osteotomy in ACL reconstruction patients with steep PTS. Data on patient-reported outcome measures, radiological parameters, knee stability as revealed by pivot shift grade, and knee anterior laxity and complications were extracted based on the inclusion criteria. The Methodological Index for Non-Randomized Study (MINORS) score was used for quality assessment. Review Manager and R statistical software were used to perform the statistical analysis.

**Results:**

Nine studies with a total of 227 knees in 91 primary and 136 revision ACLR patients were included. Nearly 60% of patients experienced ≥2 revision ACLR. In revision ACLR, the currently well-accepted indication for slope-reducing osteotomy is a slope >12° (measured on the short-segment radiographs). Primary ACLR patients with a more stringent slope (≥16°, measured on weight-bearing full-length radiographs), excessive anterior tibial translation (≥6 mm), or poor meniscus status may benefit from additional osteotomy. After combined procedures, substantial decreases were detected in the proportion of knees with pivot shift grades II–III [71/121 (58.7%) vs 19/121 (15.7%); OR = 23; *P =* 0.003] and side-to-side differential anterior laxity (weighted mean: 8.0 vs 1.9, weighted mean difference = 6.1, *P* *<* 0.00001). Significant improvements (*P* < 0.00001) were identified in the Lysholm score (weighted mean: 47 vs 82.9) and International Knee Documentation Committee score (weighted mean: 39.3 vs 71.7). The incidence of adverse events after osteotomy ranged from 0% to 39.1% with an overall pooled rate of 10.6% (95% confidence interval = 0.5%–20.7%). Hardware discomfort and graft re-rupture with high-grade pivot shift were common complications. No patients experienced surgical site infection or bone nonunion.

**Conclusion:**

Combined slope-reducing tibial osteotomy demonstrates significant improvements in both knee stability and patient-reported outcomes for revision ACLR. Similarly, primary ACLR patients with steep PTS, excessive anterior tibial translation, and poor meniscus status may derive clinical benefits from additional osteotomy.

**HIGHLIGHTS**

- Consensus has been lacking with regard to the specific indications for slope-reducing osteotomy in patients with anterior cruciate ligament (ACL) injuries.
- Combined tibial slope-correcting osteotomy demonstrates significant improvements in both knee stability and patient-reported outcomes for revision ACL reconstruction.
- Primary ACL reconstruction patients with steep posterior tibial slope, excessive anterior tibial translation, and poor meniscus status may derive clinical benefits from additional osteotomy.

## Introduction

Primary anterior cruciate ligament (ACL) reconstruction (ACLR) is a technical challenge. The number of revision surgeries following primary failure continues to increase. It has been reported that the failure rate of primary ACL reconstruction ranges from 10% to 20%^[1,2]^. The causes of graft failure can be attributed to extrinsic factors such as surgical techniques and graft selection, as well as intrinsic factors specific to the knee, including steep posterior tibial slope (PTS). In recent years, there has been growing evidence linking steep PTS to ACL graft failure.

A systematic review integrating 15 clinical studies found that patients who experienced primary reconstruction failure had steeper PTS preoperatively^[3]^. Additionally, clinical studies have shown an association between increased PTS and anterior tibial translation, which may result in increased ACL stress at anatomical positions and subsequently increase the risk of graft failure^[4–7]^. A biomechanical study has revealed that slope-reducing osteotomy greatly decreases the linear tension of the ACL graft and anterior tibial translation, potentially reducing the risk of graft failure^[8]^. Based on this evidence, some researchers advocate for the use of slope-reducing tibial osteotomy to correct steep PTS in patients with ACL injury. However, the clinical application of this concept is limited and primarily performed in revision surgeries. It is worth noting that Song *et al* applied this technique to primary ACL injury patients with concomitant chronic medial meniscus posterior horn tear and excessive anterior tibial subluxation (ATS), achieving reliable clinical outcomes^[9]^. Since Sonnery-Cottet *et al*^[10]^ proposed the use of anterior closing-wedge osteotomy to correct steep PTS clinically, various osteotomy techniques have been introduced and gradually applied in clinical practice, with corresponding reports on clinical outcomes^[11–14]^.

Although most studies included only a small number of patients and had limited data on clinical outcomes and surgical complications, the reported research data and results are highly valuable. Bosco *et al*^[15]^ and Itthipanichpong *et al*^[16]^ recently conducted systematic reviews to investigate the clinical outcomes of revision patients undergoing slope-reducing osteotomy to correct steep PTS. However, these two studies included less accurate studies, and the extraction and integration of data were not comprehensive enough, which may have resulted in less rigorous results. Additionally, a recent study reported the long-term outcomes of primary anterior cruciate ligament reconstruction (ACLR) with versus without slope-reducing osteotomy primary ^[17]^, yet these significant findings have not been systematically evaluated.

Therefore, we carried out the present systematic review and meta-analysis to investigate the clinical outcomes of slope-reducing tibial osteotomy in treating primary or revision ACLR patients with steep PTS and the appropriate surgical indications. We hypothesized that this procedure would yield satisfactory subjective and objective results in the presence of steep PTS.

## Materials and methods

This study was registered in the International Prospective Register of Systematic Reviews (PROSPERO) (registration number: CRD420251041583). This work was conducted in line with PRISMA (Preferred Reporting Items for Systematic Reviews and Meta-Analyses)^[18]^ and AMSTAR (Assessing the Methodological Quality of Systematic Reviews) Guidelines^[19]^ and TITAN (Transparency and International Transferability in Artificial Intelligence for Neuroimaging) Guideline^[20]^.

### Search strategy and eligibility criteria

The search strategy was used to identify all publications eligible for inclusion in the review. PubMed, Embase, Web of Science, and Scopus databases were searched in April 2025. The search terms are listed in Supplemental Digital Content, Appendix S1, available at: http://links.lww.com/JS9/F156 (PubMed). Specific search strategies were developed for each database, and references of the identified studies were checked for potential eligibility.

The following inclusion criteria were used to identify eligible studies: (1) publications reporting on the outcomes of slope-reducing tibial osteotomy in primary or revision ACL reconstruction and (2) patients with an average follow-up of at least 24 months. Furthermore, this study excluded non-English language reports, case reports, conference abstracts/posters, or reviews. After the removal of duplicates, two orthopedic surgeons (D.W. and T.Z.) independently reviewed the titles and abstracts to screen for potentially eligible studies. Full texts were then assessed independently by the same two reviewers to identify the final list of publications suitable for inclusion in the current study. If disagreement occurred, a third senior orthopedic surgeon (H.Z.) was consulted for final assessment and consensus.

### Data extraction

Each included article was abstracted regarding study features, patient characteristics, surgical information, and outcome measures. Study characteristics included author name, publication date, study design, level of evidence, and number of patients/knees. Patient data included sex, age, and duration of follow-up. Surgical information covered in the included studies regarding the indication of slope-reducing tibial osteotomy, osteotomy techniques, targeted PTS, and graft selection was summarized. Other additional procedures, such as lateral extra-articular tenodesis and meniscal surgery for combined injuries, were recorded. Outcome measures consisted of patient-reported outcome measures (PROMs), preoperative and postoperative PTS, pivot shift grade, and differential anterior knee laxity. PROMs included the Lysholm score, International Knee Documentation Committee (IKDC) subjective score, and Tegner activity scale (TAS). Knee arthritis and postoperative complications were also documented. The data extraction of the included studies was conducted independently by two reviewers (D.W. and T.Z.).

### Quality assessment

The quality of the included studies was assessed independently by two reviewers (D.W. and T.Z.). In this regard, the Methodological Index for Non-Randomized Study (MINORS)^[21]^ for cohort studies was used. Items on the questionnaire were scored as follows: 0 if not reported, 1 if reported but inadequate, and 2 if reported and adequate. The maximum score for non-comparative studies was 16, with a score of 13–16 being considered the low risk of bias. The maximum score for comparative studies was 24, with scores of 21 to 23 considered to be at low risk of bias. Otherwise, studies were considered to have a high risk of bias. When disagreement occurred, a third senior orthopedic surgeon (.Z.) was consulted for final consensus. The intraclass correlation coefficient (ICC) was calculated in SPSS software (version 26.0, IBM, Armonk, NY, USA) and used to evaluate the reliability of the publication bias assessment^[22]^.

### Statistical analysis

Review Manager (version 5.3 from Cochrane Collaboration) and R statistic software were used to perform the statistical analysis, with *P* < 0.05 as a threshold of statistical significance. In this study, the mean-variance estimation method described by Lou *et al*^[23]^ and Wan *et al*^[24]^ was used to estimate means (standard deviations) from median and interquartile ranges in some studies. *RevMan Calculator* was used to find the standard deviations of continuous data for studies that only reported a range of continuous variables. This tool has been created by Amy Drahota and Elaine Bellor based on the calculations provided in the Cochrane Handbook (https://training.cochrane.org/resource/revman-calculator). For continuous data with standard deviation, meta-analysis was performed to calculate the weighted mean difference (WMD) with 95% confidence intervals (CIs) using the inverse variance (IV) method. When comparing the incidence of dichotomous data, such as pivot shift grade, the odds ratio (OR) was calculated using the Mantel–Haenszel method. The *I*^2^ statistic and *Q* test were used to measure heterogeneity. If *I*^2^ < 50% and the *P*-value for the *Q* test was >0.05, the studies were interpreted as minimally heterogeneous, and a fixed-effects model was applied for the meta-analysis. A random-effects model was applied when *I*^2^ > 50% or the *P*-value for the *Q* test was <0.05, which indicated that the data were of high heterogeneity, exploring the sources of heterogeneity through subgroup analysis. R statistical software was used to calculate the occurrences of complications. Other results were presented as a descriptive summary.

## Results

### Characteristics of included studies

**Figure 1.**
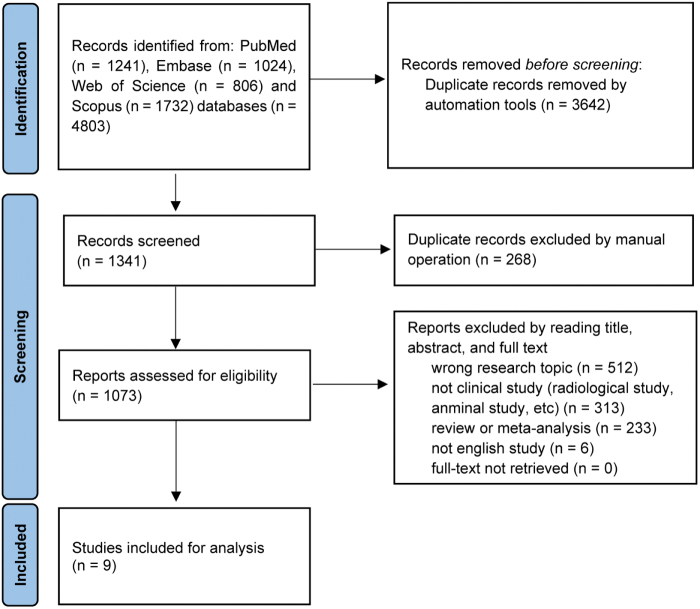
Selection of interested studies.

A total of 1341 studies were identified in the initial search, and nine articles were finally included for analysis (Fig. 1). Eight of the studies were retrospective case series without control groups, and the remaining one was a comparative study (with vs without osteotomy)^[17]^. For eight non-comparative studies, the median MINORS scores of the two reviewers were 10 (range: 8–11) and 11 (range: 8–11). Two studies were assessed as having a relatively high risk of bias (MINORS score: 8–9)^[10,11]^. The ICC for the evaluation reliability was 0.85 (95% CI: 0.14–0.98). The study features are summarized in Table 1.

**Table 1:** Characteristics of included studies and patients

Study	Design, LOE	Grouping	Patients	Knees	Age	Male: female	No. of revision (0:1:2:≥3 times)	Follow-up time	MINORS (R1/R2)
Sonnery-Cottet 2014^[10]^	Case-series, IV	Osteotomy + ACLR	5	5	24 (16–40)	4:1	0:0:0:5	31.6 (23–45) months	8/8
Dejour 2015^[11]^	Case-series, IV	Osteotomy + ACLR	9	9	30.3 ± 4.4	6:3	0:0:9:0	4.0 ± 2.0 years	9/8
Akoto 2020^[13]^	Case-series, IV	Osteotomy + ACLR	20	20	27.8 ± 8.6	14:6	0:14:5:1	30.5 ± 9.3 months	10/11
Song 2020^[9]^	Case-series, IV	Osteotomy + ACLR	18	18	29.4 (20–41)	16:2	18:0:0:0	33.2 (25–44) months	10/11
Rozinthe 2022^[25]^	Case-series, IV	Osteotomy + ACLR	9	9	39.4 ± 8.3	6:3	0:0:9:0	9.9 ± 3.0 (7–15) years	9/8
Dejour 2023^[26]^	Case-series, IV	Osteotomy + ACLR	15	15	25.3 ± 6.6	14:1	0:15:0:0	4.4 ± 1.5 years	10/11
Mabrouk 2023^[12]^	Case-series, IV	Osteotomy + ACLR	64	64	29.6 ± 6.3	53:11	≥2 times	3.0 ± 0.8 years	11/11
Vivacqua 2023^[14]^	Case-series, IV	Osteotomy + ACLR	23	23	28.7 ± 9.8	12:11	0:7:16:0	26.7 (6–84) months	11/10
Wang 2025^[17]^	Cohort design, III	Osteotomy + ACLR	25	25	29.6 ± 3.5	20:5	25:0:0:0	29.6 ± 3.5 months	21/22
		ACLR only	48	48	28.8 ± 3.4	31:17	48:0:0:0	28.8 ± 3.4 months	

LOE, level of evidence.

A total of 227 knees in 91 primary and 136 revision ACLR patients with increased PTS were included. In 179 osteotomies, 136 (76%) knees underwent one or more revision surgeries, and 100 (55.9%) knees experienced two or more revision reconstructions. Male patients were more likely to receive slope-reducing tibial osteotomy than females [77.7% (139/179) vs 22.3% (40/179)]. The mean age of patients ranged from 24 to 29.6 years. All studies had a mean follow-up time of more than 2 years. The characteristics of the patients are summarized in Table 1.

### Indications of slope-reducing osteotomy

In revision ACLR, the currently well-accepted indication for slope-reducing osteotomy is a PTS >12°^[10–14,25]^. However, in primary ACLR, a steep PTS alone is insufficient to justify osteotomy; it must be combined with other critical structural abnormalities, such as excessive tibial anterior translation^[17]^ or chronic posterior meniscal horn tears^[9]^. The threshold for PTS correction is more stringent in primary ACLR. A recent propensity score-matched study suggested that the optimal osteotomy threshold should be set at 16° (measured on weight-bearing full-length radiographs). The study concluded that for patients with PTS ≥16°, combining osteotomy with ACLR significantly reduced graft failure rates and improved control of excessive tibial anterior translation^[17]^. Details are presented in Table 2.

**Table 2:** Details of slope-reducing osteotomy and combined procedures

Study	Osteotomy technique	Osteotomy indications	Targeted PTS (°)	LET	MM procedures	LM procedures	Graft selection in ACLR
Sonnery-Cottet 2014^[10]^	Transtuberosity	Revision + PTS > 12°	8–10	2	2 resections	0	Autografts (1 PT, 1 HT, 3 QT)
Dejour 2015^[11]^	Supratuberosity	Revision + PTS > 12°	3–5	0	0	4 repairs	Autografts (8 QT, 1 HT)
Akoto 2020^[13]^	Transtuberosity	Revision + anterior knee laxity (SSD≥6 mm) + PTS > 12°	8–10	20	8 repairs + 4 resections	0	Autografts (12 QT, 7 HT, 1 BPTB)
Song 2020^[9]^	Infratuberosity	Primary + PTS > 13° + ATS > 10 mm + chronic MPHTs (>6 months)	8–10	0	11 resections + 6 repairs	8 resections + 2 repairs	Autografts (18 ST/G)
Rozinthe 2022^[25]^	Supratuberosity	Revision + PTS > 12°	3–5	0	0	4 repairs	Autografts (8 QT, 1 HT)
Dejour 2023^[26]^	Supratuberosity	Revision + PTS > 10° + or PTS≥8° + ATS > 10 mm + poor meniscus status	0–5	0	3 repairs	7 repairs + 1 resection	Autografts (15 QT/HT)
Mabrouk 2023^[12]^	Supratuberosity	Revision + PTS > 12°	4–6	46	22 repairs	25 repairs	41 autografts (22 BPTB, 19 HT) + 18 allografts
Vivacqua 2023^[14]^	Supratuberosity (19 cases) + Transtuberosity (4 cases)	Revision + PTS > 12°	3–10	6	5 resections + 4 repairs	4 resections + 5 repairs	14 autografts (9 QT, 5 BPTB) + 9 allografts
Wang 2025^[17]^	Infratuberosity	Primary + PTS ≥ 16° + ATS ≥ 6 mm	8–10	0	12 repairs + 4 resections	3 repairs + 5 resections	Autografts (22 ST/G)

ACLR, anterior cruciate ligament reconstruction; ATS, anterior tibial subluxation; BPTB, bone-patellar tendon-bone; HT, hamstring tendon; LET, lateral extra-articular tenodesis; MPHTs, meniscus posterior horn tears; PT, patellar tendon; PTS, posterior tibial slope; QT, quadriceps tendon; SSD, side-to-side difference; ST/G, semitendinosus and gracilis tendons.

### Osteotomy techniques

Slope-reducing tibial osteotomy can be performed at different levels, including supratuberosity osteotomy^[11,12,25]^, tubercle-reflecting transtuberosity osteotomy^[10,13]^, and infratuberosity osteotomy^[9,17]^. Various osteotomy techniques have been reported to yield satisfactory clinical outcomes. However, the lack of comparative studies makes it difficult to establish a clear preference among different approaches. Recent studies suggested that infratubercle osteotomy may be a more favorable option, as it avoids tibial tubercle disruption, prevents interference with graft placement, provides optimal intraoperative exposure, and allows for early weight-bearing^[9,22,27]^ (Table 2). Table 3 summarizes the pearls and pitfalls of osteotomy techniques at different levels.

**Table 3:** Summary of different slope-reducing osteotomy techniques

Osteotomy techniques	Characteristics
Supratuberosity	Technically demanding surgical exposure
	Requires careful protection of the patellar tendon during the procedure
	May influence tibial tunnel positioning in concomitant ACL reconstruction
Tubercle-reflecting transtuberosity osteotomy	Tubercle detachment potentially prolongs healing and rehabilitation
	Limited clinical adoption results in sparse outcome data
Infratuberosity	Provides superior surgical field exposure
	Increased proximal bone volume facilitates plate fixation
	Potential impact on coronal alignment
	Requires greater bone resection per degree of correction

### Evaluation of PTS

Current studies lack consensus regarding slope measurement methodologies. While some investigations employ short-segment radiographs with the proximal tibial axis for slope assessment, others utilize weight-bearing full-length images in conjunction with the tibial anatomic axis for slope evaluation (Table 4). Previous studies found that the tibial segment length used for measurement significantly impacts the obtained values^[28,29]^. In revision cases, short-segment measurement remains the predominant clinical approach. The pooled analysis showed the preoperative and postoperative PTS range from 12.5° to 18.5° (weighted mean: 14.4) and from 1.9° to 9.2° (weighted mean: 4.8), respectively (Table 4). The difference between the two statuses was significant (WMD = 9.6, 95% CI = 7.2–11.9; *P* < 0.00001).

**Table 4:** Primary and secondary outcomes of included studies

Study	PTS			Pivot test (grade 0:I:II:III)		Differential anterior knee laxity (SSD)		
	Evaluation	Pre	Post	Pre-surgery	Post-surgery	Evaluation	Pre	Post
Sonnery-Cottet 2014^[10]^	Short knee radiographs, proximal tibial axis	13.6 (13–14)	9.2 (8–10)	0:1:3:1	4:1:0:0	Telos stress device	10.4 (8–14)	2.8 (2–4)
Dejour 2015^[11]^	Short knee radiographs, proximal tibial axis	13.2° ± 2.6°	4.4° ± 2.3°	0:0:0:9	8:1:0:0	Telos stress device	11.7 ± 5.2	4.3 ± 2.5
Akoto 2020^[13]^	Short knee radiographs, proximal tibial axis	15.3 ± 11.6	8.9 ± 11.1	0:0:0:20	20:0:0:0	Rolimeter test	7.2 ± 1.3	1.1 ± 1.1
Song 2020^[9]^	Whole-leg radiographs, tibial anatomic axis	18.5 (17–20)	8.1 (7–9)	0:0:15:3	18:0:0:0	KT-1000 arthrometer	13 (10–15)	1.6 (−4 to 3)
Rozinthe 2022^[25]^	Short knee radiographs, proximal tibial axis	13.2° ± 2.6°	4.4° ± 2.3°	0:0:0:9	9:0:0:0	NA	NA	NA
Dejour 2023^[26]^	Short knee radiographs, proximal tibial axis	12.5 ± 1.8°	1.9 ± 3.6°	NA	NA	Telos stress device	4.6 ± 3.4	−4.2 ± 5.6
Mabrouk 2023^[12]^	Short knee radiographs, proximal tibial axis	13.8° ± 1.5°	4.2° ± 1.7°	15:17:22:10	22:25:14:3	KT-1000 arthrometer	7.0 ± 1.2	2.9 ± 0.8
Vivacqua 2023^[14]^	Short knee radiographs, proximal tibial axis	14 (12–18)	4 (0–15)	Grade III (6)	Grade II (2)	NA	8.5 (5.4)	3.6 (4.6)
Wang 2025^[17]^	Whole-leg radiographs, tibial anatomic axis	18.2 ± 1.9	6.7 ± 2.0	0:6:18:1	23:2:0:0	KT-1000 arthrometer	7.8 ± 2.8	1.2 ± 2.9

NA, not available; PTS, posterior tibial slope; SSD, side-to-side difference.

### Knee stability

**Figure 2.**
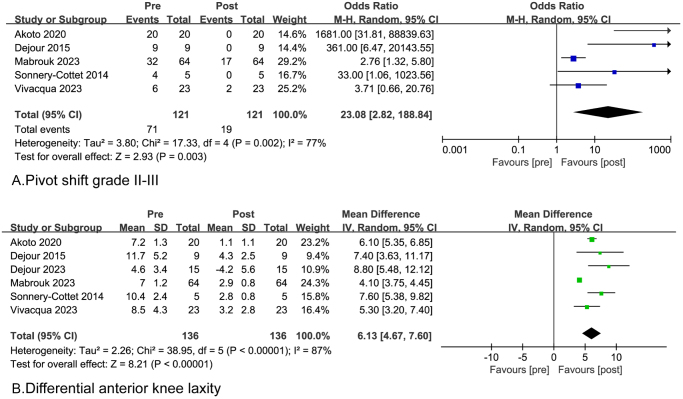
Forest plots of knee stability after the slope-reducing tibial osteotomy. (A) Pivot shift grades II–III; (B) differential anterior knee laxity.

For revision ACLR, five studies reported the preoperative and postoperative pivot shift grade and the pooled analysis showed a substantial decrease in the proportion of knees with pivot shift grades II–III after slope-reducing tibial osteotomy [71/121 (58.7%) vs 19/121 (15.7%); OR = 23; *P* = 0.003] (Table 5 and Fig. 2a). Six studies reported the preoperative and postoperative side-to-side differential anterior laxity ranging from 4.6 to 11.7 mm (weighted mean: 8.0), and from −4.2 to 4.3 mm (weighted mean: 1.9), with significant differences between the two statues (WMD = 6.1, 95% CI = 4.7–7.6; *P* < 0.00001) (Table 5 and Fig. 2b). Two articles reported pre- and post-operative results of the Lachman test in 29 patients^[11,13]^. Preoperatively, all patients had Lachman grades ≥2, while postoperatively, all knees showed negative Lachman tests, indicating good knee joint stability. For primary ACLR, combined slope-reducing tibial osteotomy demonstrates significant improvement in anterior tibial translation compared to ACLR alone (−3.1 vs −0.3 mm, *P* = 0.012), while showing no significant effect on anterior knee laxity (−6.6 vs −5.6, *P* = 0.149)^[17]^. The subgroup analysis results stratified by osteotomy techniques and surgery status are presented in Supplemental Digital Content, Appendix S2, available at: http://links.lww.com/JS9/F157.

**Table 5:** Primary and secondary outcomes of included studies

Study	IKDC		IKDC grade		Lysholm		TAS		OA grade 1:2:3		Complications
	Pre	Post	Pre	Post	Pre	Post	Pre	Post	Pre	Post	
Sonnery-Cottet 2014^[10]^	39.5 (21.8–64.4)	79.1 (48.3–98.9)	3C2D	1A4B	46.2 (26–69)	87.8 (60–100)	7.4 (5–9)	7.2 (5–9)	4:2:0	1:3:1	0
Dejour 2015^[11]^	44.1 ± 16.1	71.6 ± 6.1	5D4C	7B2C	38.4 ± 16.4	73.8 ± 5.8	NA	NA	0	1:1:0	0
Akoto 2020^[13]^	NA	NA	NA	NA	49.9 ± 21	90.9 ± 6.4	2.9 ± 1.5	6.1 ± 0.9	0	7:0:0	1 second surgery due to postoperative hematoma
Song 2020^[9]^	NA	NA	18D	14A4B	46.5 (34–58)	89.5 (78–94)	5.7 (4–6)	7.3 (6–8)	0	0	0
Rozinthe 2022^[25]^	44.1 ± 16.1	83 ± 12	NA	NA	38.4 ± 16.4	84.5 ± 11.9	NA	NA	0	0:2:1	0
Dejour 2023^[26]^	46.4 ± 17.1	80.3 ± 16.2	15D	5A10B	55.8 ± 22.5	83.8 ± 12.5	NA	Na	0	0	1 reoperation to re-suture a medal meniscal tear; 1 early superficial infection
Mabrouk 2023^[12]^	38.0 ± 12.5	69.1 ± 12.3	NA	NA	51.9 ± 14.0	74.5 ± 11.4	NA	NA	NA	NA	3 rerupture with a severe pivot shift, 11 hardware discomfort
Vivacqua 2023^[14]^	NA	52.4 ± 19.2	NA	NA	NA	NA	NA	NA	NA	NA	2 graft failure, 6 reoperation due to hardware discomfort, 1 knee instability
Wang 2025^[17]^	NA	NA	NA	NA	NA	NA	NA	NA	0	0	NA

IKDC, International Knee Documentation Committee; NA, not available;TAS, Tegner Activity Score

### Subjective outcomes

**Figure 3.**
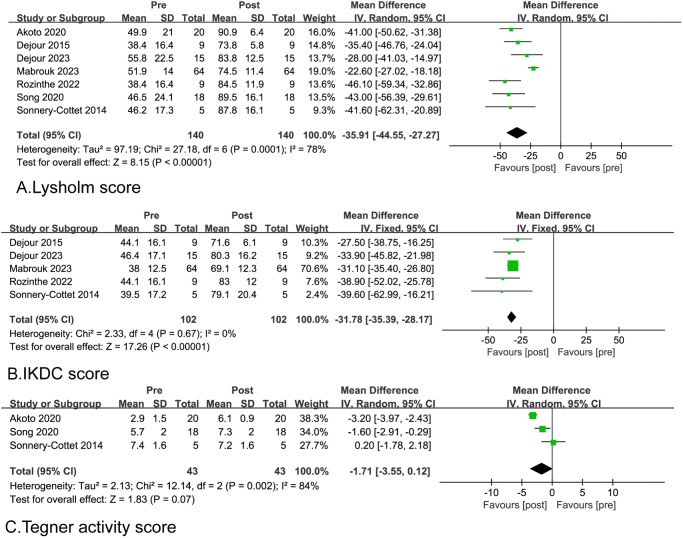
Forest plots of the patient-reported clinical outcomes after the slope-reducing tibial osteotomy. (A) Lysholm score; (B) IKDC score; and (C) Tegner activity score.

Five different PROMs were utilized among the included studies: the Lysholm score (seven studies), IKDC score (five studies), IKDC grade (four studies), Tegner score (three studies), and Knee Injury and Osteoarthritis Outcome Score (KOOS) (two studies. The pooled analysis showed the Lysholm score (weighted mean: 47 vs 82.9; WMD = −35.9, 95% CI = −44.6 to −27.3; *P* < 0.00001) and IKDC score (weighted mean: 39.3 vs 71.1; WMD = −31.8, 95% CI = −35.4 to −28.2; *P* < 0.00001). In addition, two studies reported good to excellent postoperative KOOS scores that significantly improved from the preoperative scores^[13,14]^ (Table 5 and Fig. 3).

### Complications

**Figure 4.**
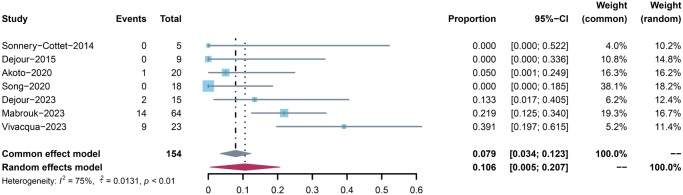
Forest plot on the incidence rates of major complications. Data pooled with a random effects model.

Four studies reported 26 adverse events in 122 procedures after slope-reducing tibial osteotomy^[9,11,12,26]^. The major complication rate of included studies ranged from 0% to 39.1% with an overall pooled risk of 10.6% (95% CI = 0.5%–20.7%) (Table 5 and Fig. 4). The most common complication is symptomatic hardware requiring reoperation [hardware discomfort/adverse cases: 17/26 (65.4%)], followed by graft re-rupture with high-grade pivot shift or trauma [5/26 (19.2%)]. No patients experienced infection or nonunion at the osteotomy site postoperatively.

## Discussion

The most important findings of this systematic review and meta-analysis were that slope-reducing tibial osteotomy can significantly reduce PTS and decrease or eliminate high-grade pivot shift and anterior knee laxity, leading to improved knee stability and subjective outcomes, along with a low graft re-rupture rate in revision and selected primary patients with ACL injury and steep PTS.

Currently, most clinical and radiographic studies suggest that steep PTS increases the risk of graft failure^[4,5,7,30]^, and this unfavorable outcome may be attributed to increased ACL tension^[31]^. Normal PTS ranges from 5° to 7°, and exceeding 12° is considered pathological. Duerr *et al*^[3]^ recently conducted a systematic review and meta-analysis including 15 relevant studies, which demonstrated that patients with primary ACL graft failure had significantly higher preoperative PTS compared to those without graft failure. Lee *et al*^[4]^ found that patients with ACL graft failure had significantly steeper PTS compared to patients with intact ACL grafts. They observed that the likelihood of ACL graft failure was 4.5 times higher in knees with PTS ≥ 12°. Christensen *et al*^[32]^ discovered that an increase in PTS was associated with increased anterior tibial translation, which may lead to increased ACL stress at anatomical positions and thereby increase the risk of ACL injury.

ACL reconstruction is a complex surgical technique. When combined with meniscal injuries, symptomatic knee instability (such as excessive anterior tibial subluxation), and structural abnormalities (such as steep PTS), the procedure becomes more challenging, and the clinical outcomes become unpredictable^[9]^. Surgical technique mistakes, particularly inappropriate tunnel position^[11]^, are widely recognized as the most common cause of revision surgery (accounting for approximately 70% of revision cases^[33]^. With the increasing popularity and precision of surgical techniques and arthroscopy, the issue of anatomical tunnel position has been greatly addressed. However, inherent factors in the knee, such as steep PTS, have not received sufficient emphasis in ACL reconstruction. Sonnery-Cottet *et al*^[10]^ suggested that in patients with repeated ACL revisions, the position of the graft tunnel is relatively reliable, and in such cases, steep PTS becomes an important risk factor for graft failure. This also explains why anterior closing wedge osteotomy techniques are often applied to patients with recurrent graft failures.

Current evidence suggests that slope-reducing tibial osteotomy combined with ligament reconstruction represents a reliable indication for failed ACL reconstruction cases with increased PTS, particularly in those with PTS > 12°^[3,11,12,14,34]^. It should be noted that this study measured PTS using the whole leg radiograph, which tends to yield results approximately 2° higher compared to the short knee radiographs used in previous studies^[10–12,25,35,36]^. However, whether this osteotomy procedure should be applied during primary ACLR remains controversial. Wang *et al* recently demonstrated that concomitant osteotomy effectively reduces postoperative ATS and graft laxity in primary ACLR patients, with greater clinical benefits observed in cases exhibiting higher PTS angles^[17]^. Their proposed 16° (measured with full-length lower limb radiography) is the optimal threshold for osteotomy in primary ACLR.

Biomechanical studies indicate that increased PTS increases anteroposterior shear stress on the meniscus, potentially exacerbating ATS^[37,38]^. This supports the notion that steep PTS serves as a shared risk factor for ACL tears, excessive ATS, and meniscal horn tears. Therefore, for primary ACL injuries combined with steep PTS, excessive ATS, and MPHTs, slope-reducing tibial osteotomy may represent a more reliable treatment option.

When performing primary ACL reconstruction, we need to consider whether a steep PTS affects the anatomical tunnel position of the graft. Recent studies have shown a significant correlation between steep PTS and excessive anterior tibial subluxation (ATS) in extension (>10 mm) after ACL injury^[31]^. Grassi *et al* suggested that in such cases, achieving proper tunnel positioning becomes a dilemma: placing the tunnels anatomically can cause impingement of the graft^[30]^. Song *et al* also demonstrated that anatomical ACL reconstruction alone may not restore excessive ATS^[39]^. Therefore, if excessive ATS is not restored, to avoid the impingement of the graft, the tibial tunnel needs to be posteriorly placed. However, the posterior tunnel position will be considered “non-anatomical,” especially when the excessive ATS is restored, which becomes another form of “surgical technique mistake” and can lead to higher failure rates in ACL reconstruction. Given this, we need to consider whether correcting steep PTS first and then proceeding with anatomical graft tunnel position during primary ACL reconstruction can effectively reduce the occurrence of graft failure. Song *et al*^[9]^ propose that in 18 cases with steep PTS, chronic meniscal posterior horn tears, and excessive ATS, performing slope-reducing tibial osteotomy combined with primary ACL reconstruction resulted in good joint stability and subjective scores for all patients. This suggests that tibial osteotomy could be a reliable solution in specific primary ACL patients.

There are three main surgical techniques for correcting excessive PTS: tibial deflexion osteotomy proposed by Dejour *et al*^[11]^, anterior closing-wedge osteotomy described by Sonnery-Cottet *et al*^[10]^, and the technique by Hees^[40]^. One important issue was the management of the tibial tubercle. Tibial deflexion osteotomy is performed above the tibial tubercle without detaching the patellar tendon, but the osteotomy site may interfere with the tibial tunnel and result in a slight patellar alta. The anterior closing-wedge osteotomy technique proposed by Sonnery-Cottet *et al* requires detachment of the patellar tendon^[10]^. In the technique reported by Wang^[17]^, the osteotomy site is located at the distal end of the tibial tubercle, without detaching the patellar tendon and without interfering with the tibial tunnel. Strong fixation methods allow effective rotation control and enable early weight-bearing postoperatively, speeding up the recovery process. None of the included studies in this article reported serious surgical technique-related complications or symptomatic limitation of range of motion (hyperextension within 5° being the most common, but without symptoms).

This study showed that, for revision cases, combined osteotomy significantly reduces postoperative pivot-shift grading and improves anterior knee stability (KT-1000 side-to-side difference) and patient-reported outcomes. However, due to the lack of control groups, it remains challenging to assess the precise contribution of isolated osteotomy to these clinical effects based on the available studies. Notably, the most recent comparative study demonstrated that^[17]^, although tibial osteotomy further reduced anterior tibial translation in primary ACL reconstruction, the improvement in anterior knee stability was not greatly significant. In addition, there currently exists no direct clinical evidence comparing the effects of these three surgical techniques on knee joint stability. Based on current clinical experience, supratuberosity osteotomy may compromise the accuracy of tibial tunnel positioning, while infratuberosity osteotomy could potentially alter lower limb alignment. Nevertheless, the clinical significance of these effects requires further validation through additional data. These findings suggest that the stabilizing effect of slope-reducing tibial osteotomy on knee stability remains inconclusive.

Yamaguchi *et al* have demonstrated that slope-reducing tibial osteotomy significantly decreases ACL tension while simultaneously altering knee kinematics^[8]^. Under axial loading conditions, slope correction affects anterior tibial translation in both medial and lateral compartments. Since this procedure does not modify the coronal or rotational alignment of the tibia relative to the femur, the osteotomy does not influence tibial internal rotation torque or varus/valgus torque during daily loading conditions, as confirmed by mechanical testing^[8]^. A recent radiographic study^[41]^ revealed that slope correction can impact coronal plane alignment, inducing a mean varus change of 1°. Expert consensus suggests that coronal malalignment exceeding 5° should be avoided when performing slope correction^[42]^. Therefore, the study concludes that standardized surgical techniques – particularly adequate exposure and balanced osteotomy – can prevent clinically significant alterations in coronal plane mechanics.

This study has limitations. First, the search strategy contained bias due to the possibly unavoidable omission of relevant studies. However, four main databases were searched to include all the relevant studies reporting the clinical outcomes following the slope-reducing tibial osteotomy procedure. Second, considerable heterogeneity was observed when comparing pre- versus postoperative clinical outcomes, necessitating cautious interpretation of the pooled results. Third, the exclusive inclusion of retrospective studies and paucity of direct comparisons between combined osteotomy and isolated ACL reconstruction significantly constrained the evidence level of this meta-analysis. Future investigations should prioritize prospective comparative studies to better elucidate the long-term effects of tibial osteotomy and various osteotomy techniques on clinical outcomes. Finally, although this study utilized standardized statistical methods to convert raw data (expressed as ranges) into standard deviations for data integration, it may potentially affect the weighting of data and the pooled outcomes in meta-analysis.

## Conclusion

The slope-reducing tibial osteotomy can significantly reduce PTS and decrease or eliminate high-grade pivot shift and anterior knee laxity, leading to improved knee stability and subjective outcomes, along with a low graft re-rupture rate in revision and selected primary patients with ACL injury and steep PTS.

## Supplementary Material

## Data Availability

Data yielded in our study will be made available by the authors to any qualified researchers.

## Research registration unique identifying number (UIN)

We registered our study in the international prospective register of systematic reviews (PROSPERO) (CRD420251041583) and conducted this systematic review. Link: https://www.crd.york.ac.uk/PROSPERO/view/CRD420251041583.
