# Supplementary material for: Slope-reducing tibial osteotomy combined with primary or revision ACL reconstruction improves knee stability and subjective function in patients with steep posterior tibial slope: a systematic review and meta-analysis
Source: Int J Surg. 2025 Sep 19;112(1):1855–64. doi: 10.1097/JS9.0000000000003507 (PMC12825645; doi:10.1097/JS9.0000000000003507)
Supplement: Supplementary file 1 [file js9-112-1855-001.docx]

**Table 1** The search strategy used in this study (PubMed)

| Search | Query | Results |
| --- | --- | --- |
| #1 | "anterior cruciate ligament"[MeSH Terms] OR ("anterior"[All Fields] AND "cruciate"[All Fields] AND "ligament"[All Fields]) OR "anterior cruciate ligament"[All Fields] OR "ACL"[All Fields] | 41,481 |
| #2 | "Osteotomy"[MeSH Terms] OR "osteotom*"[All Fields] OR "correct*"[All Fields] | 914,213 |
| #3 | "tibia"[MeSH Terms] OR "tibia*"[All Fields] OR "slope*"[All Fields] OR "posterior*"[All Fields] OR "PTS"[All Fields] | 600,403 |
| #4 | #1 AND #2 AND #3 | 1,241 |

Original search: 2025-04-04, updated data: 2025-04-23.
