# Supplementary material for: Slope-reducing tibial osteotomy combined with primary or revision ACL reconstruction improves knee stability and subjective function in patients with steep posterior tibial slope: a systematic review and meta-analysis
Source: Int J Surg. 2025 Sep 19;112(1):1855–64. doi: 10.1097/JS9.0000000000003507 (PMC12825645; doi:10.1097/JS9.0000000000003507)
Supplement: Supplementary file 2 [file js9-112-1855-002.pdf]

Study or Subgroup	Pre Events	Pre Total	Post Events	Post Total	Weight	Odds Ratio M-H, Random, 95% CI
**8.1.1 Transtuberosity**						
Akoto 2020	20	20	0	20	14.6%	1681.00 [31.81, 88839.63]
Sonnery-Cottet 2014	4	5	0	5	16.7%	33.00 [1.06, 1023.56]
**Subtotal (95% CI)**		**25**		**25**	**31.3%**	**206.69 [4.43, 9649.64]**
Total events	24		0			
Heterogeneity: Tau² = 4.14; Chi² = 2.16, df = 1 (P = 0.14); I² = 54%						
Test for overall effect: Z = 2.72 (P = 0.007)						
**8.1.3 Supratuberosity**						
Dejour 2015	9	9	0	9	14.4%	361.00 [6.47, 20143.55]
Mabrouk 2023	32	64	17	64	29.2%	2.76 [1.32, 5.80]
Vivacqua 2023	6	23	2	23	25.2%	3.71 [0.66, 20.76]
**Subtotal (95% CI)**		**96**		**96**	**68.7%**	**6.04 [1.07, 34.08]**
Total events	47		19			
Heterogeneity: Tau² = 1.41; Chi² = 5.63, df = 2 (P = 0.06); I² = 65%						
Test for overall effect: Z = 2.04 (P = 0.04)						
**Total (95% CI)**		**121**		**121**	**100.0%**	**23.08 [2.82, 188.84]**
Total events	71		19			
Heterogeneity: Tau² = 3.80; Chi² = 17.33, df = 4 (P = 0.002); I² = 77%						
Test for overall effect: Z = 2.93 (P = 0.003)						
Test for subgroup differences: Chi² = 2.70, df = 1 (P = 0.10), I² = 62.9%						

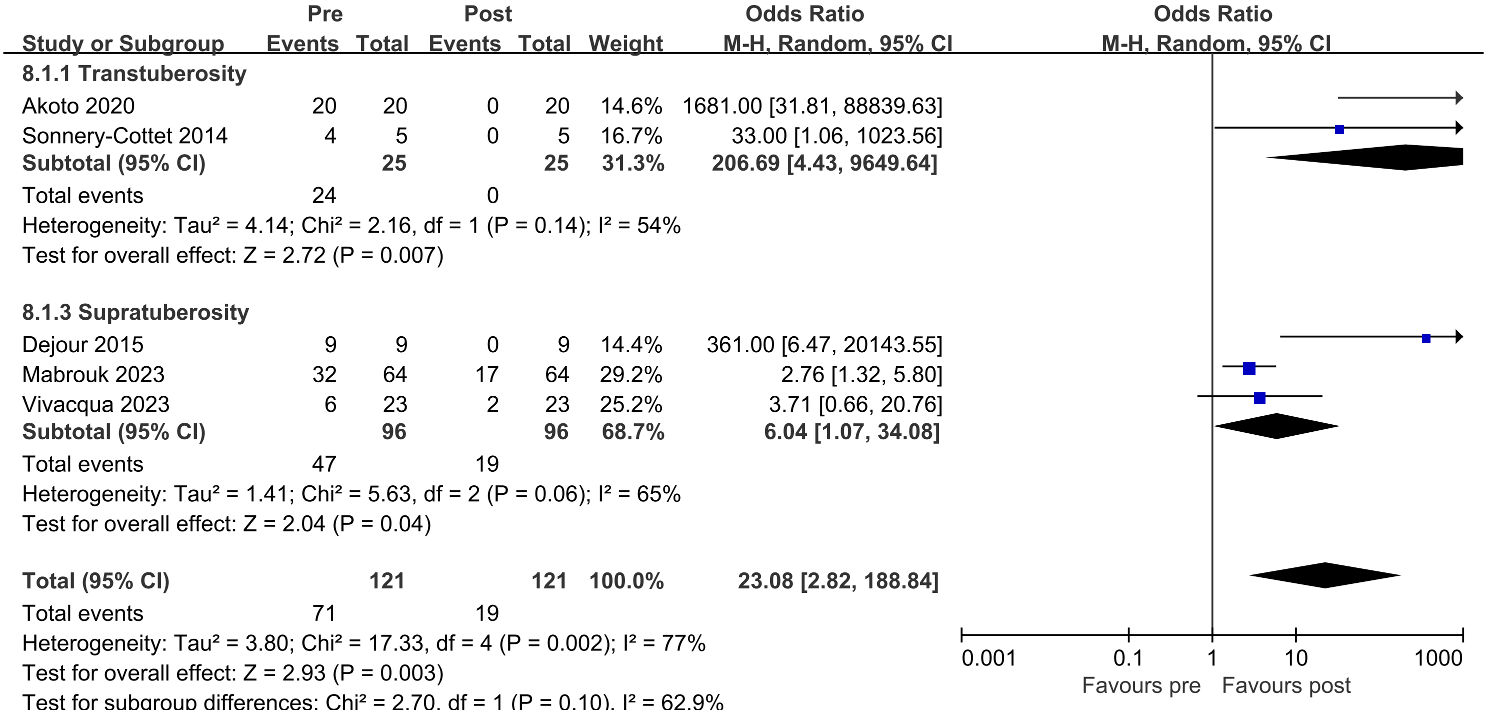

Appendix2-1-Pivot shift grade II-III-Subgroup analysis based on the osteotomy techniques

Study or Subgroup	Pre Events	Pre Total	Post Events	Post Total	Weight	Odds Ratio M-H, Random, 95% CI
**9.1.1 Revision pts**						
Akoto 2020	20	20	0	20	11.7%	1681.00 [31.81, 88839.63]
Dejour 2015	9	9	0	9	11.6%	361.00 [6.47, 20143.55]
Mabrouk 2023	32	64	17	64	19.7%	2.76 [1.32, 5.80]
Sonnery-Cottet 2014	4	5	0	5	13.1%	33.00 [1.06, 1023.56]
Vivacqua 2023	6	23	2	23	17.8%	3.71 [0.66, 20.76]
**Subtotal (95% CI)**		**121**		**121**	**73.9%**	**23.08 [2.82, 188.84]**
Total events	71		19			
Heterogeneity: Tau² = 3.80; Chi² = 17.33, df = 4 (P = 0.002); I² = 77%						
Test for overall effect: Z = 2.93 (P = 0.003)						
**9.1.2 Primary pts**						
Song 2020	18	18	0	18	11.7%	1369.00 [25.77, 72720.79]
Wang 2025	19	25	0	25	14.5%	153.00 [8.12, 2883.29]
**Subtotal (95% CI)**		**43**		**43**	**26.1%**	**331.84 [31.29, 3518.97]**
Total events	37		0			
Heterogeneity: Tau² = 0.00; Chi² = 0.79, df = 1 (P = 0.38); I² = 0%						
Test for overall effect: Z = 4.82 (P < 0.00001)						
**Total (95% CI)**		**164**		**164**	**100.0%**	**55.25 [6.84, 446.27]**
Total events	108		19			
Heterogeneity: Tau² = 5.62; Chi² = 31.64, df = 6 (P < 0.0001); I² = 81%						
Test for overall effect: Z = 3.76 (P = 0.0002)						
Test for subgroup differences: Chi² = 2.73, df = 1 (P = 0.10), I² = 63.4%						

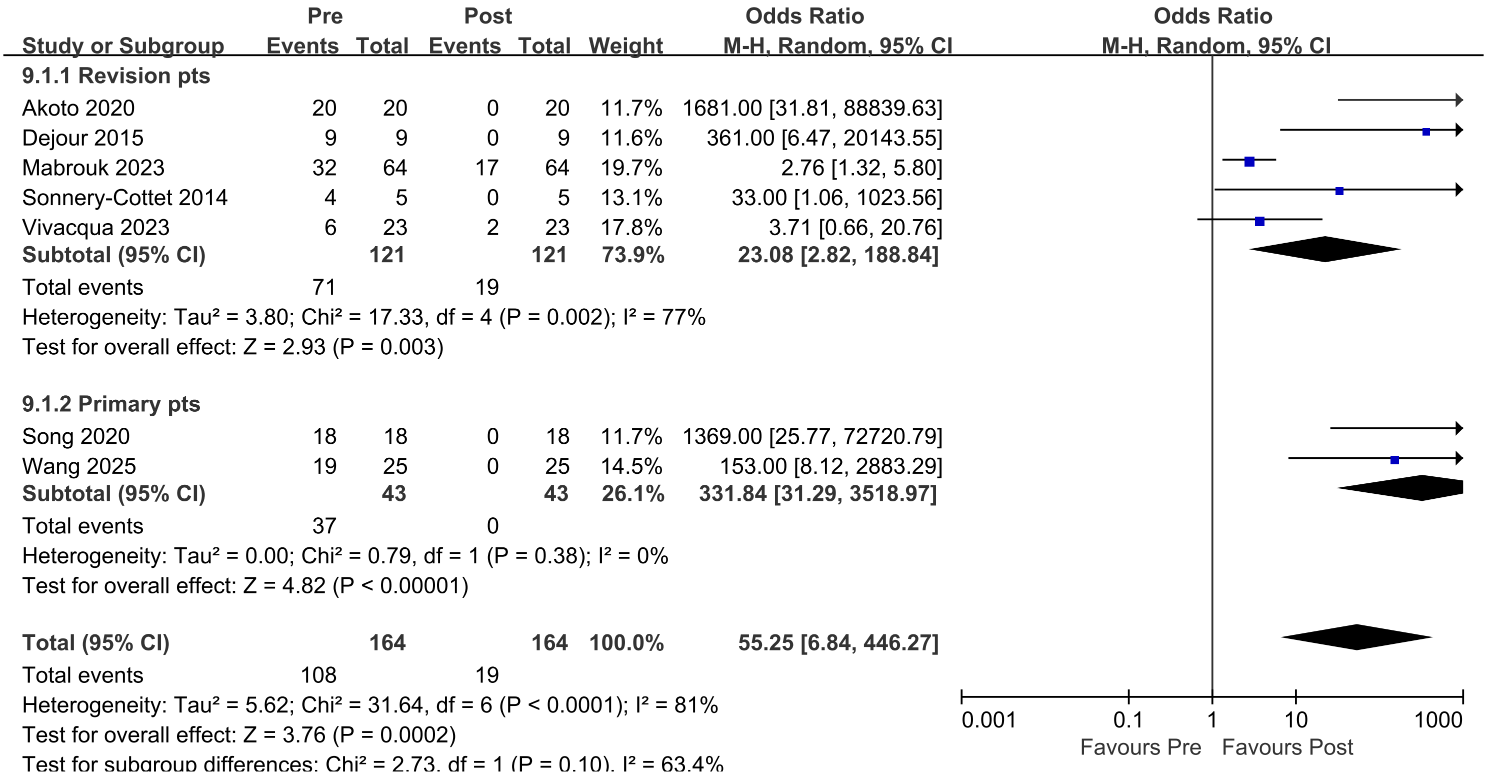

Appendix2-2-Pivot shift grade II-III-Subgroup analysis based on the revision or primary surgery

	Pre			Post				Mean Difference
**Study or Subgroup**	**Mean**	**SD**	**Total**	**Mean**	**SD**	**Total**	**Weight**	**IV, Random, 95% CI**
**10.1.1 Transtuberosity**								
Akoto 2020	7.2	1.3	20	1.1	1.1	20	23.2%	6.10 [5.35, 6.85]
Sonnery-Cottet 2014	10.4	2.4	5	2.8	0.8	5	15.8%	7.60 [5.38, 9.82]
**Subtotal (95% CI)**			**25**			**25**	**39.0%**	**6.47 [5.20, 7.74]**
Heterogeneity: Tau² = 0.41; Chi² = 1.58, df = 1 (P = 0.21); I² = 37%								
Test for overall effect: Z = 9.99 (P < 0.00001)								
**10.1.2 Supratuberosity**								
Dejour 2015	11.7	5.2	9	4.3	2.5	9	9.4%	7.40 [3.63, 11.17]
Dejour 2023	4.6	3.4	15	-4.2	5.6	15	10.9%	8.80 [5.48, 12.12]
Mabrouk 2023	7	1.2	64	2.9	0.8	64	24.3%	4.10 [3.75, 4.45]
Vivacqua 2023	8.5	4.3	23	3.2	2.8	23	16.4%	5.30 [3.20, 7.40]
**Subtotal (95% CI)**			**111**			**111**	**61.0%**	**5.87 [3.81, 7.94]**
Heterogeneity: Tau² = 2.95; Chi² = 11.52, df = 3 (P = 0.009); I² = 74%								
Test for overall effect: Z = 5.58 (P < 0.00001)								
**Total (95% CI)**			**136**			**136**	**100.0%**	**6.13 [4.67, 7.60]**
Heterogeneity: Tau² = 2.26; Chi² = 38.95, df = 5 (P < 0.00001); I² = 87%								
Test for overall effect: Z = 8.21 (P < 0.00001)								
Test for subgroup differences: Chi² = 0.24, df = 1 (P = 0.63), I² = 0%								

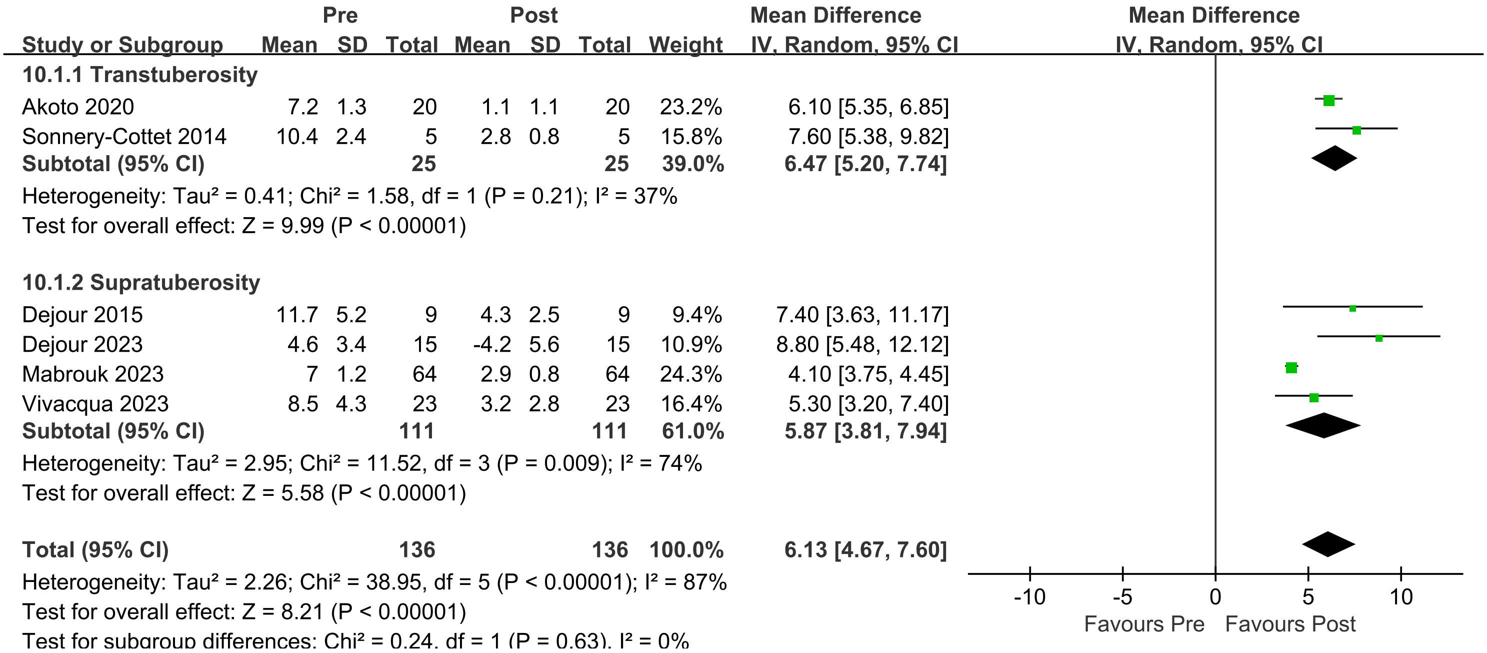

Appendix2-3-Differential anterior knee laxity-Subgroup analysis based on the osteotomy techniques

Study or Subgroup	Pre Mean	Pre SD	Pre Total	Post Mean	Post SD	Post Total	Weight	Mean Difference IV, Random, 95% CI
**11.1.1 Revision pts**								
Akoto 2020	7.2	1.3	20	1.1	1.1	20	15.8%	6.10 [5.35, 6.85]
Dejour 2015	11.7	5.2	9	4.3	2.5	9	8.2%	7.40 [3.63, 11.17]
Dejour 2023	4.6	3.4	15	-4.2	5.6	15	9.3%	8.80 [5.48, 12.12]
Mabrouk 2023	7	1.2	64	2.9	0.8	64	16.3%	4.10 [3.75, 4.45]
Sonnery-Cottet 2014	10.4	2.4	5	2.8	0.8	5	12.2%	7.60 [5.38, 9.82]
Vivacqua 2023	8.5	4.3	23	3.2	2.8	23	12.5%	5.30 [3.20, 7.40]
**Subtotal (95% CI)**			**136**			**136**	**74.2%**	**6.13 [4.67, 7.60]**
Heterogeneity: Tau² = 2.26; Chi² = 38.95, df = 5 (P < 0.00001); I² = 87%								
Test for overall effect: Z = 8.21 (P < 0.00001)								
**11.1.2 Primary pts**								
Song 2020	13	5	18	1.6	1	18	11.8%	11.40 [9.04, 13.76]
Wang 2025	7.8	2.8	25	1.2	2.9	25	14.0%	6.60 [5.02, 8.18]
**Subtotal (95% CI)**			**43**			**43**	**25.8%**	**8.92 [4.22, 13.62]**
Heterogeneity: Tau² = 10.47; Chi² = 11.00, df = 1 (P = 0.0009); I² = 91%								
Test for overall effect: Z = 3.72 (P = 0.0002)								
**Total (95% CI)**			**179**			**179**	**100.0%**	**6.91 [5.38, 8.44]**
Heterogeneity: Tau² = 3.71; Chi² = 75.63, df = 7 (P < 0.00001); I² = 91%								
Test for overall effect: Z = 8.86 (P < 0.00001)								
Test for subgroup differences: Chi² = 1.23, df = 1 (P = 0.27), I² = 18.5%								

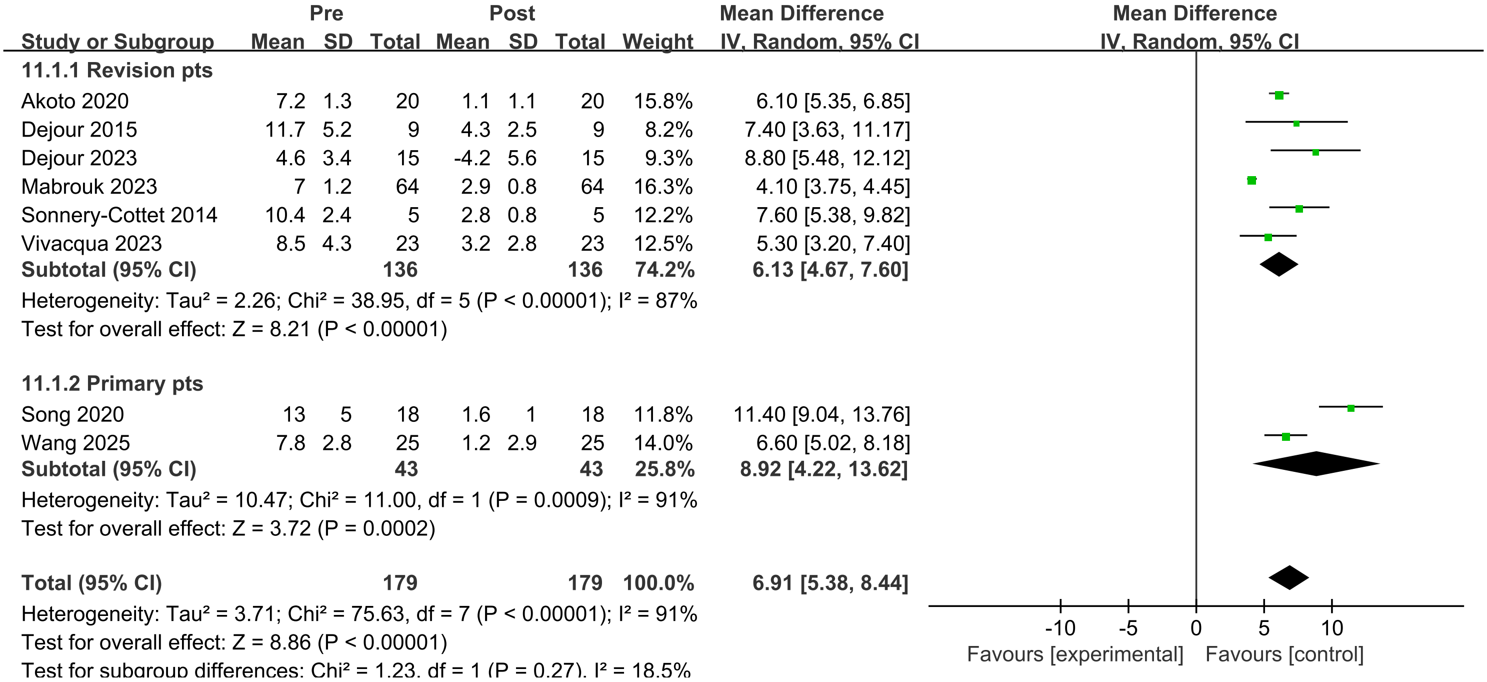

Appendix2-4-Differential anterior knee laxity-Subgroup analysis based on the revision or primary surgery
